# Chain length-dependent luminescence in acceptor-doped conjugated polymers

**DOI:** 10.1038/s41598-019-47537-2

**Published:** 2019-08-02

**Authors:** Pieter van der Scheer, Ties van de Laar, Joris Sprakel

**Affiliations:** Physical Chemistry and Soft Matter, Wageningen University & Research, Stippeneng 4, 6708WE Wageningen, The Netherlands

**Keywords:** Physical chemistry, Polymer chemistry

## Abstract

Semiconducting polymers doped with a minority fraction of energy transfer acceptors feature a sensitive coupling between chain conformation and fluorescence emission, that can be harnessed for advanced solution-based molecular sensing and diagnostics. While it is known that chain length strongly affects chain conformation, and its response to external cues, the effects of chain length on the emission patterns in chromophore-doped conjugated polymers remains incompletely understood. In this paper, we explore chain-length dependent emission in two different acceptor-doped polyfluorenes. We show how the binomial distribution of acceptor incorporation, during the probabilistic polycondensation reaction, creates a strong chain-length dependency in the optical properties of this class of luminescent polymers. In addition, we also find that the intrachain exciton migration rate is chain-length dependent, giving rise to additional complexity. Both effects combined, make for the need to develop sensoric conjugated polymers of improved monodispersity and chemical homogeneity, to improve the accuracy of conjugated polymer based diagnostic approaches.

## Introduction

Conjugated polymers are a versatile class of building blocks combining the properties of a polymer and a semi-conducting material. Mostly used for making advanced optical and opto-electronic materials such as photovoltaics^1,2^, OLED’s^3,4^ and insulated molecular wires^5,6^, displays, memories, batteries. In addition to their value in creating physical optoelectronic devices and active layers, conjugated polymers have recently emerged as a valuable platform to build molecular diagnostic tools in solution. These approaches rely on the effectuation of conformation changes of the conjugated polymer, e.g. by analyte binding or mechanical stretching, and the resulting alteration of the luminescence pattern. The relationship between luminescence emission and chain conformation is complex, depending on a variety of factors, including the chemical composition, presence of chromophores, chain length, solubility and intermolecular interactions. For conjugated homopolymers, these effects have, e.g., been harnassed to measure viral capsid formation, where encapsulation and subsequent stretching of the conjugated polymer lead to distinct changes in the vibronic fine structure^7^, or to probe various types of analyte binding or enzymatic action by means of superquenching^8,9^. Also more complex architectures employing conjugated homopolymers, such as polydiacetylene vesicles, have been used to detect species in solution based on changes in fluorescence emission resulting from conformational changes in the semiconducting polymer^10–12^.

The optical response to changes in the spatial conformation of a polymer chain can be tailored and amplified through the doping of the semiconducting chain with a minor fraction of energy transfer acceptors, positioned as chromophores along the chain. Both the excitonic transfer along the semiconducting backbone, as well as the Förster resonant energy transfer through the dielectric medium within the polymer coil, change substantially as the chain goes from a solvated and coiled conformation to one that is either stretched or collapsed. For example, extensive work in the group of Bazan has shown the sensitive, and sequence-specific, detection of small amounts of DNA based on the binding-induced conformational changes in acceptor-doped polyfluorenes^13–15^. A similar approach has been used to evaluate nanoscopic structural changes in self-assembled nanostructures during electrostatic condensation^16^. More recently, our group has shown that mechanical tension can induce a gradual transition from coil to stretched chain, that can be optically deduced down to the scale of single molecules^17^, leading to their use as highly sensitive molecular tension sensors.

Most synthetic methods to produce semiconducting polymers yield rather polydisperse products. Yet, it is well established that the equilibrium chain conformation, and its sensitivity to changes in response to an external cue, depend strongly on their chain length. This poses the question how these sensory approaches based on semiconducting polymers suffer from chain polydispersity, and how the sensitivity of the measurement could potentially be tuned if the chain length can be well controlled. For several prototypical conjugated homopolymers, including polyfluorenes^6,18–20^, polythiophenes^21–24^, polyacetylene^23,25^, phenylenevinylene^22,26,27^, phenyleneethynylenes^28,29^, effects of polymerisation degree on their optoelectronic properties has been established. For example, for polyfluorenes, systematic synthesis of pure oligomers has lead to a detailed insight in how the luminescence of these polymer changes with increasing conjugation length. Above a chain length of approximately 6 repeat units, no further changes in emission patterns were observed, thereby defining the maximum conjugation length in extended chains^18,19^. While emission properties stop changing when the polymerisation degree exceeds the conjugation length, structural properties such as the glass transition and isotropization temperature continue to evolve^19^.

By contrast, very little is known about the chain length effects on the emission patterns of conjugated polymers that are doped with a minority fraction of chromophores, despite its importance in further developing conjugated polymer-based solution diagnostics. By contrast to homopolymers, chain length effects in doped chains have both conformational and compositional origins. Due to the semiflexible backbone of many conjugated polymers, very short chain lengths result in rigid rod behavior, with little to no conformational flexibility. Indeed, previous work has shown that doped chains below their conjugation length are not effective as molecular sensors^17^. The conformational freedom of the chain continues to grow with increasing chain length, thereby increasing the sensitivity by which changes in conformation, e.g. due to analyte binding or mechanical tension, can be interrogated optically. An additional degree of complexity in minority-doped conjugated chains, which often feature a random incoorporation of the chromophore during synthesis, is the heterogeneity of the chemical composition, even if the chain length would be perfectly homogeneous. The probabilistic nature of chromophore incorporation, and the resulting variations in chemical composition between chains, results in strong variations of energy transfer efficiency within a population. We have previously shown that while acceptor-doped donor polymers can be used as ultrasensitive tension sensors, chain length has a substantial effect on the tension-optical response curve. This offers the opportunity to tune the mechano-optical response of these molecular sensors by chain length, but simultaneously highlights how polydispersity increases the experimental uncertainty in these optically-based mechanical tension assays. To increase the reliability and sensitivity of solution-based sensory platforms based on acceptor-doped conjugated polymers, it is crucial that we develop new approaches to improve the chain length and composition homogeneity, and understand which factors influence chain length dependencies in the optical response.

In this paper, we set out to understand the mechanisms of chain length dependent emission patterns in acceptor-doped semiconducting polymers. To do so, we fractionate polydisperse conjugated polymer reaction products into series of fractions of well-defined molecular weight using solvent-gradient Soxhlet extractions. Spectroscopic analysis of fractions of donor polymers, doped with a minority fraction of acceptors, shows a strong chain length dependence in the intrachain energy transfer. We explain these results, aided by computer simulations, as the result of the binomial distribution of acceptor incorporation that ensues from the probabilistic acceptor incoorporation during the polymerisation. Furthermore, we perform this analysis on two different chemical designs, to highlight that our findings are not specific to a single chemical design, and may be generic to a much wider class of minority-doped semiconducting polymers.

## Results

**Figure 1:**
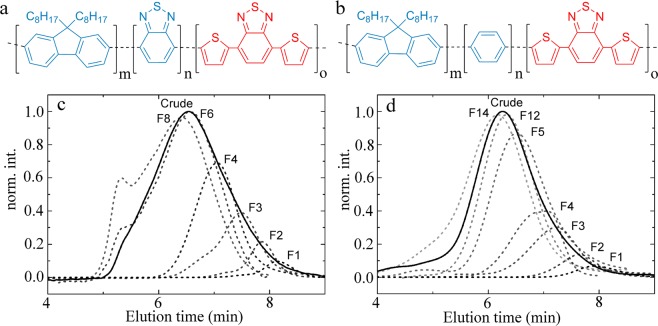
(**a**) Chemical structure of poly(dioctylfluorene-alt-benzothiadiazole)-co-(dithienyl benzothiadiazole) (*P*1), with m = 7, n = 6, o = 1. (**b**) Chemical structure of poly(dioctylfluorene-alt-benzene)-co-(dithienyl benzothiadiazole) with m = 6, n = 7, o = 1 (*P*2). (**c**,**d**) Gel permeation chromatography elution traces for *P*1 (**c**) and *P*2 (**d**). The crude product is drawn as a solid line, different subfractions in dashed lines as indicated.

We explore two different chemical designs for a donor polymer doped with a small fraction of energy transfer acceptors. The first polymer (*P*1) features a green emitting backbone, composed of alternating segments of dioctyl fluorene and benzothiadazole (BT), that together form an effective green emitting donor moiety, as the energy transfer between fluorene and BT has an efficiency close to unity. The second polymer (*P*2) features a donor backbone composed of alternating dioctylfluorene and phenyl moeities, which emits in the blue. Both polymers are doped by introducing a minority fraction of dithienyl benzothiadiazole (DTBT), as a blue/green absorbing and red-emitting acceptor chromophore. The chemical structure of these two designs is illustrated in Fig. 1a,b. These designs are based on previous studies in which these architectures have been successfully used as molecular tension sensors^17^, inspired by previous work in the group of Bazan on DNA detection^13,14^.

The synthesis of such doped semiconducting polymers often occurs through carbon-carbon coupling polycondensations, such as Suzuki or Yamamoto polymerizations^30–36^, in which both chain length and chemical composition of the polymers is ill-controlled. Methods for controlled polymerizations of semiconducting polymers, e.g. Kumada polymerizations^37–41^, are not readily ammenable to synthesize complex doped copolymers. As a result, the cleaned but crude product features a broad size distribution, from which it is impossible to evaluate the effects of individual fractions of chain length on the overal optical properties.

We use solvent-gradient Soxhlet extraction, used previously on both conjugated and non-conjugated polymers^42–45^, to fractionate the polydisperse crude mixture into fractions of increasing lenth with improved monodispersity. The normalized gel permeation chromatography elution traces are shown in Fig. 1c,d; these data illustrate that we obtain fractions spanning a rather broad range of molecular weights with improved polydispersity with respect to the starting product. This approach gives us access to a set of fractions of increasing average molecular weight (SI Fig. 1a,b), all derived from the same synthesis reaction, and thus featuring identical *average* chemical composition. Proton NMR spectra of a short and long chain fraction of P1 are shown in the Supporting Information; we observe a distinct peak broadening for the longer chains due to slower molecular relaxations. The different monomers appear as a clustered set of peaks in the aromatic region; combined with the peak broadening, this makes quantitative assignement more challenging. Hence, we turn to spectroscopic means to evaluate the structure of the chains as a function of their molecular weight.

### Chain-length dependent spectroscopy

**Figure 2:**
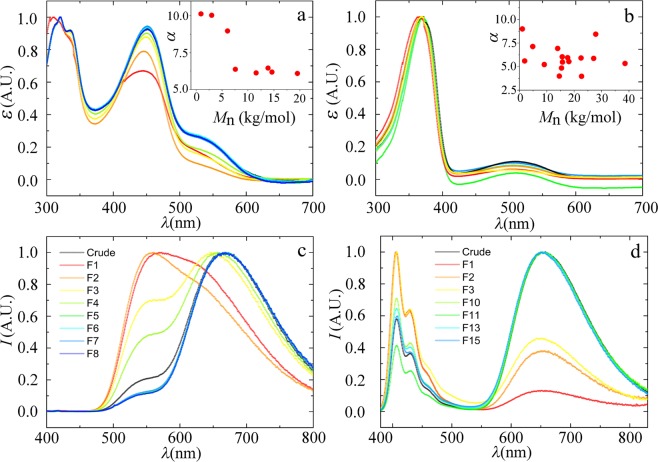
(**a**,**b**) Normalised UV-VIS absorption spectra for several fractions of *P*1 (**a**) and *P*2 (**b**). Inset show the ratio *α* of integrated donor to acceptor absorption intensity, defined as $\alpha ={\int }_{370}^{500}\,I(\lambda )/{\int }_{500}^{700}\,I(\lambda )$ for *P*1 (**a**) and $\alpha ={\int }_{300}^{420}\,I(\lambda )/{\int }_{420}^{700}\,I(\lambda )$ for *P*2 (**b**). (**c**,**d**) Corresponding normalized fluorescence emission spectra, upon donor excitation at *λ*_*ex*_ = 370 *nm*, for several fractions of *P*1 (**c**) and *P*2 (**d**).

Now that we have obtained a systematic set of fractions of the same polymer at different lengths, we can explore how chain length affects the optical properties of these acceptor-doped conjugated polymers, which is the main aim of this study. The UV-VIS absorption spectra, as shown in Fig. 2a,b, show distinct absorption bands for each of the different chromophores within the chain. By comparing the integrated intensities of donor and acceptor moieties, we can evaluate the ratio of donor and acceptor groups in the chemical composition of the polymer across the different fractions (see insets in Fig. 2a,b). For polymer *P*2 we find a homogeneous chemical composition across all fractions, as the donor-to-acceptor intensity ratio *α* is noisy but constant, and the spectra show no systematic variations (Fig. 2b). By contrast, for *P*1, we find that the chemical composition itself shows a chain length dependency, as the ratio *α* is much larger for short chain lengths, i.e. the shorter chains are enriched in donor, as compared to the higher *M*_*w*_ fractions. This is most likely the result of a composition-dependent solubility of short chains, where the presence of one strongly aromatic DTBT acceptor, which does not carry solubilizing alifatic tails, has a pronounced effect on the solubility of the entire oligomer. This result illustrates the first contribution to chain-length dependent emission patterns in doped semiconducting polymers, which is a chain-length dependent chemical composition, that can emerge during polymer purification. For both polymers we do not observe substantial shifts in the wavelength of maximal absorption *λ*_*max*_, which indicates that all fractions feature chains with degrees of polymerization that exceed the conjugation length. For polyfluorene homopolymers it is known that the conjugation length constitutes approximately 6–7 repeat units, below which the addition of each additional monomer shifts both the absorption maximum and molar exctinction coefficient^18,19^. Although the spectra as shown in Fig. 2a,b, are normalized, we did not observe significant or systematic changes in the molar exctinction between any of the fractions studied.

In previous studies, conformation-sensitive changes in donor-to-acceptor energy transfer have been used to deduce information about the spatial configuration of the semiconducting chains, e.g., to detect binding of DNA, proteins or block copolymers^13,16,46^. To probe how these effects are chain length dependent, we record the fluorescence emission of solutions of our fractionated polymers upon donor excitation at *λ*_*ex*_ = 370 nm.

The fluorescence spectrum of *P*1 shows donor emission, from the BT moeities, at *λ* = 550 nm and acceptor emission from the DTBT acceptors at 680 nm, for all fractions (Fig. 2c). It is immediately clear, however, that the relative amount of acceptor emission, indicative of the efficiency of energy transfer within chains, grows with increasing length. One could speculate that this is due to the previously observed change in chemical composition in *P*1 with chain length. However, we also observe the same effect for *P*2, which is chemically more homogeneous; this polymer exhibits donor emission peaked at 415 nm, showing distinct vibronic transitions in the donor emission band, characteristic for polyfluorenes, and acceptor emission at 650 nm. Also for *P*2 we observe a distinct increase in acceptor emission with increasing chain length. This suggests that the chain-length dependent luminescence is not only due to changes in chemical composition, but must have additional origins as well.

**Figure 3:**
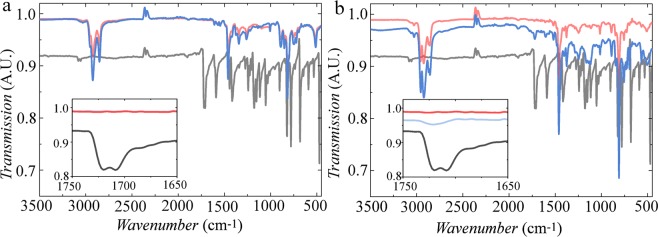
(**a**) Transmission IR spectra of *P*1 − *F*1 & *P*1 − *F*7 in red and blue & dibromo fluorenone in black. Same data from 1750–1650 cm^−1^ inset (**b**) Same as in a but for *P*2 − *F*2 & *P*2 − *F*13.

To confirm that these effects are not due to chemical defects in the chain, we realise that the most common defect in polyfluorene-based semiconducting polymers is the oxidative keto-impurity that forms from mono-alkylated fluorenes and exhibits distinct green emission^47–49^. We perform infrared spectroscopy experiments on several polymer fractions (see Fig. 3a,b) and compare these to a reference material which is pure fluorenone. These results show that the characteristic keto-stretching mode at 1721 cm^−1^ is virtually absent in all fractions. This implies that the observed differences in the fluorescent properties of both polymers are not due to chemical-defects.

We also note that chain-end defects cannot be at the root of the observed *M*_*w*_ effects; one of the two polymers was carefully end-capped during the synthesis to mitigate these potential defects, while the other polymer was not. In both cases, we observe the same behaviour, thus excluding the role of chain-ends in the length-dependent emission patterns.

Finally, we note that these chain-length dependent effects are most pronounced in solution. A main application domain of semiconducting polymers is the formation of solid-state optoelectronic devices. The luminescence spectra in the solid state, as shown in the SI, give near complete energy transfer for both short and long chains. This is due to a dominant role for interchain energy transfer. We do note a distinct red-shift of the spectra in the solid state with increasing chain length. This has been previously observed in different semiconducting polymers and attributed to the formation of intramolecular stacks^50^. In solution, this pathway is suppressed and only intrachain effects emerge. Due to the recent interest in semiconducting polymers for solution-based sensoric applications, we turn our focus to the intrachain effects alone.

### Time-correlated single-photon counting

To explore the origin of these effects, we turn to measurements of the lifetime of the excited state of the donor. The donor lifetime $\tau$ is a very sensitive measure for the energy transfer efficiency to acceptor moieties in the same chain; energy transfer leads to a proportional decrease in $\tau$, making lifetime measurements the method of choice to quantify energy transfer efficiencies.

**Figure 4:**
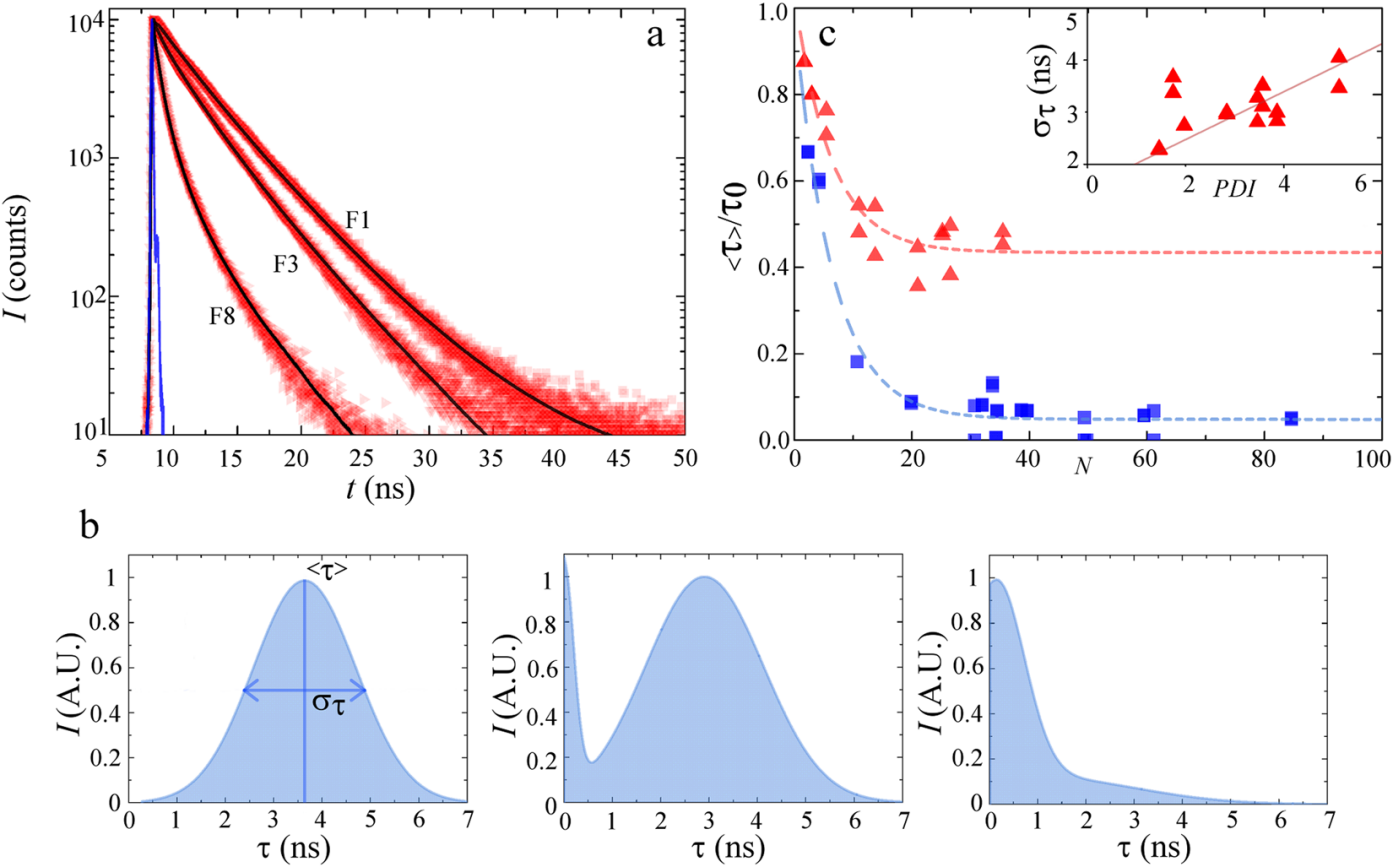
(**a**) Time-resolved fluorescence measurements of the donor in *P*1, upon excitation at *λ* = 372 nm, and detection at 550 nm. Instrument response function indicated as a solid line (blue). Black lines are fits to these spectra to obtain the lifetime distributions, as shown in (**b**). (**c**) Normalized mean donor fluorescence lifetime as a function of chain length, expressed as the number of repeat units *N*, for all *P*1 (red triangles) and *P*2 (blue squares). Inset shows the width of the lifetime distributions ${\sigma }_{\tau }$, as a function of polydispersity (PDI) for *P*1.

We study the chain-length dependence of the donor lifetime for the same fractions as studied in Fig. 2. The time-resolved fluorescence decays (Fig. 4a) show a clear decrease in donor lifetime with increasing chain length, indicating an increase in energy transfer efficiency, in line with the spectral observations in Fig. 2. We deconvolve the time-resolved fluorescence decay curves by fitting them to a series of exponential decays to obtain the excited state lifetime distributions, as shown in Fig. 4b. All fractions exhibit a rather broad lifetime distribution. We speculate that this is due to the internal remaining polydispersity index (PDI) within each fraction. To elucidate this point, we measure the width ${\sigma }_{\tau }$ of the donor lifetime distributions as the full-width at half maximum. We indeed find a positive, linear, correlation between the width of the chain length distribution and the width of the donor life distribution for polymer *P*1. This suggests that the lifetime of the donor backbone itself is dependent on its length and spatial conformation.

To analyse the chain-length dependent energy transfer, we measure the mean donor lifetime from the distributions (Fig. 4c) as a function of degree of polymerisation *N*. Since the absolute donor lifetimes vary as a function of the chemical design of the backbone, we plot these data normalized to ${\tau }_{0}$, which is the extrapolated donor lifetime in the limit of $N\to 0$, to reflect the relative change in $\tau$ as a function of chain length. Interestingly, we find for both polymers a significant decrease in the donor lifetime, and thus increase in energy transfer efficiency, with increasing chain length for short chains, reaching a steady-value for chains above a critical chain length *N*, which is approximately *N* = 15 units for *P*1 and 20 units for *P*2, as shown in Fig. 4b.

Clearly, there is a strong chain-length dependent emission in these acceptor-doped conjugated polymers, but this effect is only present for chains below a critical chain length. We have previously shown that the intramolecular FRET efficiency in these systems is a measure for the segmental density in the polymeric coil^17^. However, the fact that the chain length effect on the emission is only present for sufficiently short chains, suggests that an additional effect must be at play.

The synthesis of these co-polymers is a probabilistic process, in which the addition of monomers to growing and extending chains, is essentially random. The probability of attaching an acceptor monomer as the next addition to the chain is only determined by the ratio of donor and acceptor monomers in the reaction mixture. The number of acceptors *n*_*A*_ per individual chain is dictated by a binomial distribution, which depends only on the chain length *N* and the molar fraction of acceptors in the reaction mixture *p*, which is *p* = 0.14 for our experiments. The binomial distribution assumes that the selection of monomers in the sequence of *N* trials is independent, which we presume to hold in the early stages of the reaction where the reaction is well below conversion and an ample supply of unreacted monomers of both species is available.

The probability of finding *n*_*A*_ acceptors in a chain of length *N*, with an average acceptor fraction *p* set by the reaction mixture, is:

1 $$P({n}_{A})=((\begin{array}{c}N\\ {n}_{A}\end{array})){p}^{{n}_{A}}{(1-p)}^{N-{n}_{A}}.$$

The consequence of this binomial acceptor distribution across the chains is that, especially for small *N*, a significant fraction of chains will not have any acceptors, set by:

2 $$P({n}_{A}=0)={(1-p)}^{N}.$$

In these chains, the energy transfer efficiency is $E\equiv 0$ by definition. We hypothesize, that it is this fraction of acceptor-free chains that biases the energy transfer efficiency to lower values than dictated by the mean doping degree, and results in the distinct chain length dependency we observe. This hypothesis is supported by previous single-molecule experiments, in which we recorded the fluorescence spectra of very long and very short doped conjugated polymers. Indeed, these data appear to hint at a significant fraction of zero-acceptor chains for the short oligomers, while this fraction is virtually absent for the longer chains^17^.

### Simulations

To verify our hypothesis, we employ computer simulations to explore the role of the incorporation distribution on the intrachain energy transfer. We use Brownian Dynamics simulations to generate thermodynamic conformations for semiflexible bead-spring chains of length *N*, and use a theoretical approach to compute the intrachain energy transfer efficiency from these conformations (see Materials and Methods and ref.^17^ for details).

Because the physical dimensions of both donor and acceptor monomers are approximately 1 nm, the coarse-grained approach of modelling the chain as a strand of identically-sized beads is reasonable. In the assignment of acceptor beads along the chain, we chose a random positioning, given one constraint, that is that two acceptors can never occupy adjacent beads. This is physically realistic due to the reaction mechanism employed in the polymer synthesis. In a Suzuki-Miyaura polycondensation, aromatic bromides react with boronic acids to form a direct C-C bond. Since the acceptors are di-bromo-functional, there can never be a direct coupling between two of these units.

**Figure 5:**
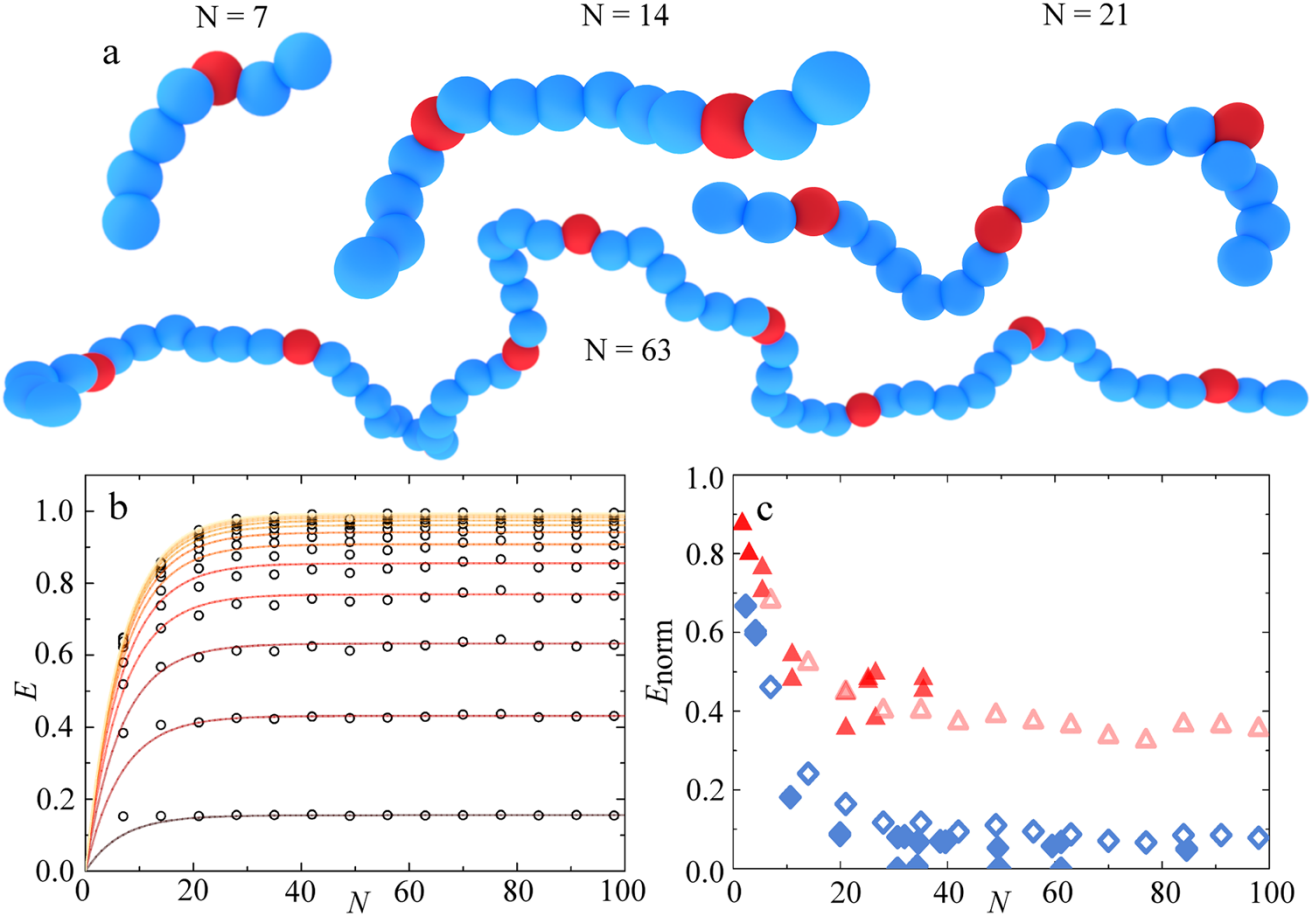
(**a**) Computer-generated renderings of typical chain conformations with donor (blue) and acceptor (red) distributions from the BD simulations for (left-to-right) *N* = 7,14, 21, 63 (**b**) Calculated energy transfer efficiency *E* (symbols) as a function of chain length *N* with increasing increasing *r*_*f*_, ranging from *r*_*f*_ = 1 nm (bottom) to *r*_*f*_ = 10 nm (top). Solid lines are predictions based on the binomial distribution, as explained in the text. (**c**) Normalized *E*, as explained in the text, for experimental data (filled symbols) and simulations (open symbols), for both *P*1 (red) and *P*2 (blue).

These simulations, that account for the binomial acceptor distribution indeed capture the chain length dependence also observed in our experiments. The energy transfer efficiency *E* grows with increasing chain length *N* up to a critical chain length beyond which the resulting optical properties remain virtually constant (Fig. 5c). Interestingly, this effect is very sensitive to the Förster radius *r*_*F*_. The fraction of zero-acceptor chains has a *E* that is zero by definition. If the average energy transfer efficiency is low, due to a small *r*_*F*_, the effect of this *E* = 0 population is modest, while for chains in which a substantial amount of energy is transferred to acceptors, when *r*_*F*_ is large, their effect is strong.

To describe these data, we realise that the ensemble-average value of $\bar{E}$, as measured in experiments, is an average of the the acceptor-free chains, which have *E* = 0, and the acceptor-rich chains. Thus, the measured $\bar{E}$ can be expressed as:

3 $$\bar{E}=E({n}_{A} > 0)\ast (1-{(1-p)}^{N}))$$

in which *E*(*n*_*A*_ > 0) is the average transfer efficiency of all chains but those lacking an acceptor and the second term the overall probability of chains having *n*_*A*_ > 0. The limiting plateau value of $\bar{E}$ at high chain lengths, reflects the value in the absence of acceptor-free chains. Thus, *E*(*n*_*A*_ > 0) is this plateau value, that can be directly deduced from these computations. Indeed, this form accurately describes the observed changes in $\bar{E}$ with N, as shown from the drawn lines in Fig. 5b.

Finally, we can compare our experimental data to these simulations results directly. Given that our simulations are parametrized to match the conformational flexibility and dimensions of the experimental polymers, the only unknown is the Förster radius *r*_*F*_, that sets the efficiency of the energy transfer between donors and acceptors through the dielectric solvent. To enable a comparison, we plot the experimental and theoretical data as $\frac{1-E}{1-{E}_{0}}=\frac{\tau }{{\tau }_{0}}$. By adjusting the value of *r*_*F*_, we can fit the experiments and find the effective value for the Förster radius. Interestingly, the agreement between the simulations, simple theoretical description, and experimental results is excellent. This supports the hypothesis that the binomial acceptor incorporation is at least partly responsible for the observed chain-length dependencies.

It is important to note, as we will also evidence in the following section, that this simulation model is highly coarse-grained and only considers the effects of chemical inhomogeneity across a population of chains on the energy transfer. Yet, additional effects, e.g. those related to excitonic migration may very well be chain length dependent as well.

Furthermore, this allows us to measure the Förster radius indirectly. We find a good agreement between simulations and experiments for *r*_*f*_ = 4.3 nm for P1; this is close to the value previously measured at 4.9 nm for this design^17^. For *P*2 we find *r*_*f*_ = 7.3 nm; this appears high and may hint at the presence of secondary effects not captured by our coarse-grained model.

### Streak imaging

**Figure 6:**
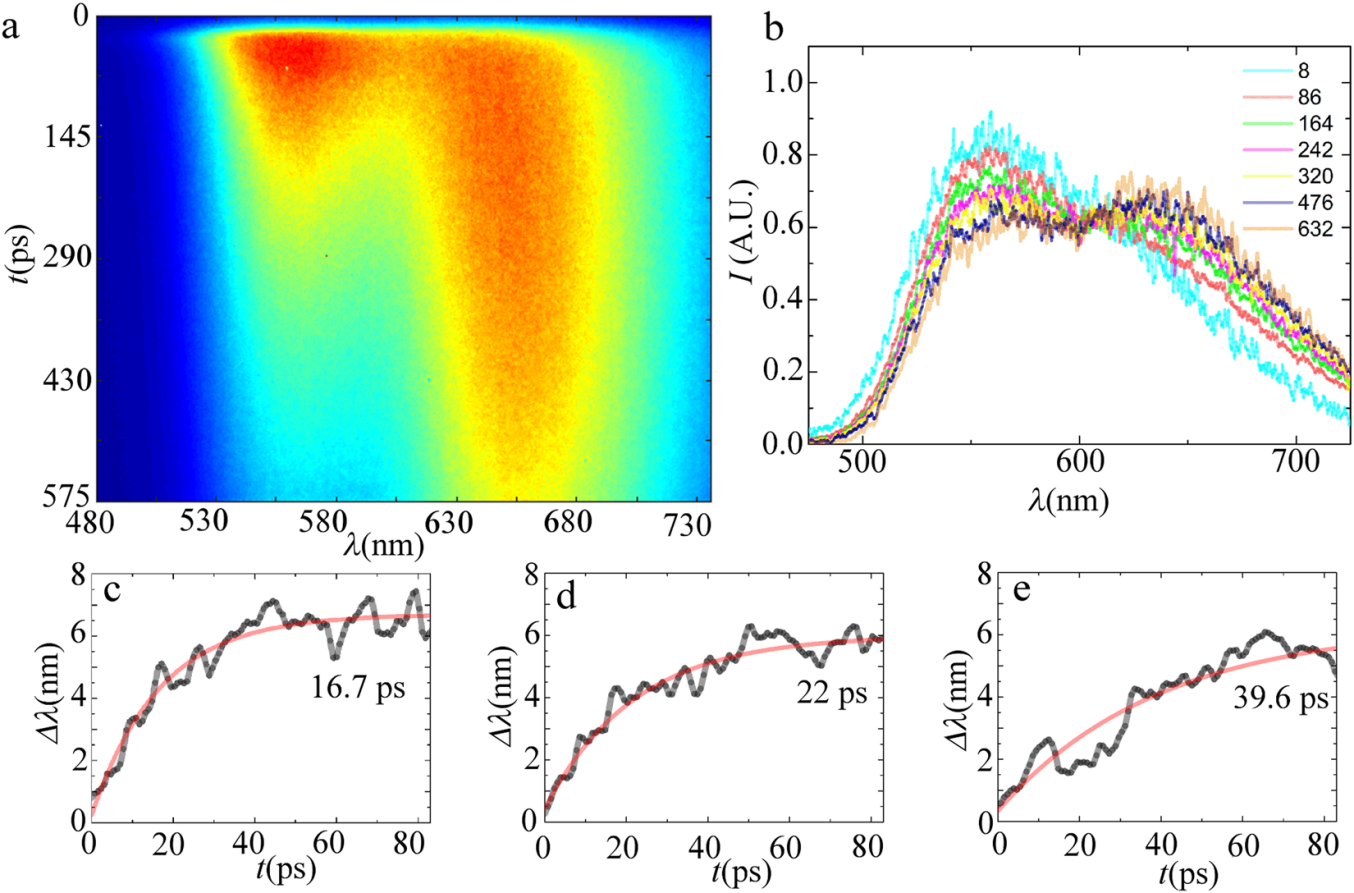
(**a**) STREAK image of *P*1 − *F*8 in CHCl_3_, (**b**) corresponding time-resolved emission spectra at selected timepoints as indicated in the legend (time in ps), (**c**,**d**) initial donor redhshift as an indicator for exciton migration for *P*1 − *F*2 (**c**), *P*1 − *F*3 (**d**) and *P*1 − *F*8 (**e**).

Finally, to evaluate if chain length also effects the rate of excitonic migration processes along the chain we perform streak imaging experiments on dilute solutions of our polymer *P*1, such that interchain excitonic transfer is suppressed. The sample is excited with a pulse of a *λ* = 385 nm laser source; an example of a resulting time-resolved fluorescence streak image is shown in Fig. 6a.

The donor-acceptor nature of our chains gives rise to an initial donor emission, as shown in time resolved spectra in Fig. 6b, that gradually gives way to acceptor emission, over the course of several hundreds of picoseconds to nanoseconds through energy transfer. The slow rate of this process hints at a predominance of Förster type transfer. Exciton migration in these polymers is established to manifest by a distinct redshift of the donor emission peak with time^51,52^. For each time point, we spectrally deconvolve the fluorescence spectra to identify the peak position of the donor band. Indeed we observe the temporal red shift of the donor peak that is a signature of exciton migration phenomena (see Fig. 6c–e). We find that these data are reasonably well described by an exponential function, from which we can extract a characteristic lifetime ${\tau }_{ex}$ for the exciton migration process. For the longest polymer, *P*1 − *F*8 with *N*_*w*_ = 75 kg/mol, we find ${\tau }_{ex}=39.6\,{\rm{ps}}$, which is in agreement with values reported in literature^51,53,54^. Also here, we observe a distinct chain length dependency; the excitonic lifetime is substantially shorter for low *M*_*w*_ chains, e.g. reducing to ${\tau }_{ex}=16.7\,{\rm{ps}}$ for *F*2 with *M*_*w*_ = 2.4 kg/mol and ${\tau }_{ex}=22.0\,{\rm{ps}}$ for *F*3 with *M*_*w*_ = 5.9 kg/mol of the same polymer. The shortening of the excitonic migration rate with chain length was also observed in solid films of similar polymers^51,55^. This effect can be explained by considering exciton migration as a one-dimensional diffusion process along individual chains. The excitonic polaron diffuses along the linear chain until it reaches a chain end where it reacts and annihilates^55^. Within this picture, the characteristic lifetime for excitonic migration should grow quadratically with diffusion length and thus with molecular weight of the chain. This is in qualitative agreement with our results.

These results illustrate that the chain length dependent emission patterns we observe are due to a combination of the chemical inhomogeneity resulting from the binomial acceptor incorporation during polymer synthesis and from inherent chain-length effects on excitonic processes along the semiconducting chains.

## Discussion

Doped conjugated polymers have emerged in the past years as attractive platforms to build molecular sensors, capable of detecting a wide variety of solution analytes, ranging from heavy metal ions to complex biomolecules^13^, illuminating complex self-assembly processes^7,16,46^ and enabling the optical detection of miniscule mechanical forces^17^. The inherent polydispersity that results from conjugated polymer synthesis procedures, reduces the accuracy with which signals can be quantitatively interpreted and complicates the direct comparison with theoretical models and simulations. This is particularly problematic when the optical read-out of the molecular sensor is performed at low numbers, or even at the scale of single molecules^17,56–58^, where large chain length dependencies of the optical response combined with strong polydispersity can be very disadvantageous, especially since the nature of chain length effects remain incompletely understood. In this paper we have used a sets of fractionated conjugated polymers, doped with a minority fraction of acceptor chromophores, to explore the origins of strong length-dependent emission patterns and to shed light on the role of chain polydispersity on the fidelity of sensoric approaches based on these molecular systems.

On the basis of our experiments and computer simulations, we conclude that this is caused in large part by the presence of a finite fraction of chains that do not carry even a single acceptor, due to the binomial statistics of acceptor build-in in the polymerisation reaction. We also showed that chain-length dependent exciton migration rates play a role as well in giving rise to spectral changes with alterations in the molecular weight of these doped polymers.

Removing the main source of chain-length dependent optical properties, which is a particularity of conjugated polymers doped with a minority fraction of acceptors, requires to ensure all chains carry at least one acceptor, or ideally that the acceptor positioning within the chain is well-controlled. Sequence-controlled polymerisation methods have seen a surge in development in the past year for non-semiconducting polymers^59–63^, yet these methods remain to be explored to create sequence-controlled conjugated polymers, where efforts have been limited to the synthesis of well-defined conjugated oligomers^18,19^, that are well-below the length where the interesting coupling between chain conformation and optical response emerges.

## Methods

### p(F8-BT-DTBT) (P1)

Conjugated polymers composed primarily of donor moieites, and doped with a minority fraction of acceptors, are prepared via standard Suzuki-Miyaura polycondensation reactions^7,17,46,64–66^. For poly(dioctyl fluorene - alt - benzothiadiazole - co - dithienyl benzothiadiazole) (P1) we dissolve 10 g (17.90 mmol, 7 equivalents) 9,9-dioctylfluorene-2,7-diboronic acid bis (1,3-propanediol) ester, 1.17 g (2.56 mmol) 4,7-Bis(5-bromo-2-thienyl)-2,1,3-benzothiadiazole and 4.51 g (15.34 mmol) 4,7-Dibromobenzo[c]-1,2,5-thiadiazole in 350 ml toluene and add 150 ml 2 M K_2_CO_3_ under nitrogen atmosphere. After thorough deoxygenation, we add 524 mg (0.716 mmol) [1,1′-Bis(diphenylphosphino) ferrocene]dichloropalladium(II). The reaction is performed for 96 hours at 100 °C. The crude product is obtained by precipitation in cold methanol and Soxhlet extraction against methanol and acetone. Yield: 80% (mol/mol), 9.56 g. *P1*-*Fraction 8*: *1H NMR* (*600* *MHz*, *CDCl3*) *δ*: 8.318 (s), 8.106 (s), 8.05 (s), 8.006–7.886 (m), 7.859–7.816 (m), 7.804–7.666 (m), 7.581–7.52 (m), 2.161 (s) 1.28–1.088 (m), 1.012–0.872 (m), 0.8430.782 (t).

*13C NMR* (*600* *MHz*, *CDCl3*) *δ*: 154.2, 151.69, 140.79, 136.34, 133.44, 128.29, 127.89, 123.88, 119.93, 55.46, 40.12, 31.76, 30.04, 29.22, 23.89, 22.58, 13.97.

### p(F62-B-DTBT) (P2)

Using the same method, we prepare poly(dioctyl fluorene - alt - benzene - co - dithienyl benzothiadiazole) (P2), using the following amounts: 5.42 gr (9.88 mmol, 6 equivalents)) 9,9-Di-(2-ethylhexyl)-2,7-dibromofluorene, 3.80 g (11.53 mmol, 7 equivalents) 1,4-benzene diboronic acid bis(pinacol) ester, 756.04 mg (1.65 mmol, 1 equivalents)4,7-Bis(5-bromo-2-thienyl)-2,1,3-benzothiadiazole and 250 mg [1,1′-Bis(diphenylphosphino) ferrocene] dichloropalladium(II). This polymer was endcapped after 48 h of reacting, by the addition of 300 mg phenylboronic acid, and reacting for another 2 h, followed by the injection of 2 ml of de-oxygenized bromobenzene and reacting for another 12 h. Yield = 78% (mol/mol), 4.22 g. %.

*P1*-*Fraction 4C*: *1H NMR* (*400* *MHz*, *CDCl3*) *δ*: 7.90–7.74 (m), 7.73–7.61 (m), 2.13 (s), 1.47–1.41 (m),1.30–1.18 (m), 0.91 (s), 0.68 (s), 0.59 (s).

*13C NMR* (*400* *MHz*, *CDCl3*) *δ*: 151.35, 140.58, 127.57, 125.96, 122.48, 120.10, 55.21, 44.58, 34.75, 33.92, 30.33, 28.29, 27.17, 22.77, 14.00, 10.40.

### Fractionation

Complete details on the different fractions, including the extraction solvent, molecular weight and polydispersity, are provided in the SI.

### GPC

Samples were prepared by dissolving 5–10 mg of polymer in 2 ml of chromatography-grade chloroform. The samples were heated gently to aid dissolution, and left to dissolve overnight under continuous agitation. All samples were filtrated over a 0.2 micron PTFE syringe filter prior to injection into the column. Measurements are performed at a chloroform flux of 1 ml/minute at 35 °C. Molecular weights were calibrated against PS standards, and corrected to compensate for higher rigidity of the polyfluorenes as compared to the standards^67^.

### Optical spectroscopy

All optical spectroscopy experiments are performed in chloroform solutions, containing 5 *μ*g/ml of polymer, placed in a quartz cuvette after filtering over a 0.2 micron PTFE syringe filter. Absorption spectra are recorded on a Shimadzu UV-2600. All fluorescence excitation, emission and lifetime measurements were performed on an Edinburgh FS5, equipped with a 372 nm pulsed laser and Time-Correlated Single-Photon Counting module for lifetime measurements. All depicted spectra are an average of two consecutive measurements on duplicate samples. Infrared transmission measurements were performed in the solid state on a Bruker Tensor 27 spectrometer. Time-resolved fluorescence measurements were performed with a streak camera setup (Hamamatsu model C5680 with model M5675 Synchroscan unit) at room temperature. Fs pulses were generated with a repetition rate of 75.9 MHz using a laser system (Coherent Mira 900F) to generate light at 770 nm. The repetition rate was reduced to 3.8 MHz by a puls picker (APE Berlin). The excitation wavelength was made by frequency doubling (APE Berlin) the output of the Ti:sapphire laser. The laser power was reduced to 30 mW (with a focal spot of 150 m). Measurements were performed at room temperature within a 800 ps time-window from 480–730 nm.

### Simulations

To rationalise our experimental findings, we complement the measurements by in-silico predictions of the chain-length dependence of the intrachain Förster energy transfer in acceptor-doped conjugated donor polymers. We begin by computing thermodynamic polymer chain conformations, using Brownian Dynamics simulations, performed in HOOMD-Blue^68,69^. We model our polymers as semiflexible bead-spring chains in a good solvent with a degree of polymerisation *N*, using a modified version of the Kremer-Grest model^70^ that includes a bending penalty to impart semiflexibility^71,72^.

All beads along the chain interact by the steeply repulsive Weeks-Chandler-Anderson potential:

4 $${U}_{WCA}\{\begin{array}{ll}4\varepsilon [{(\frac{\sigma }{r})}^{12}-{(\frac{\sigma }{r})}^{6}]+C({r}_{cut}) & r\le {r}_{cut}\\ 0 & r > {r}_{cut}\end{array}$$

in which *σ* is the particle diameter, *r* the distance between two particles, *r*_*cut*_ the cut-off distance and *C*(*r*_*cut*_) a vertical shift factor, which using *r*_*cut*_ = 2^1/6^ and *C*(*r*_*cut*_) = 1 gives a purely repulsive potential, and $\varepsilon$ sets the energy scale. Bonded beads interact via a finitely-extensible nonlinear elastic (FENE) spring:

5 $${U}_{FENE}\{\begin{array}{ll}\frac{-\,k{r}_{{\max }}}{2}\,\mathrm{ln}[1-{(\frac{r}{{r}_{{\max }}})}^{2}] & r\le {r}_{{\max }}\\ \infty  & r > {r}_{{\max }}\end{array}$$

where *k* is the spring constant and *r*_*max*_ set the maximum spring extension, here fixed as *r*_*max*_ = 1.5*σ*. To model the semiflexibility of the polyfluorenes we study in this paper, we also include a bending penalty between adjacent bonds:

6 $${U}_{bend}({\theta }_{ijk})=\kappa [1-\,{\cos }({\theta }_{ijk})]$$

where *θ*_*ijk*_ is the bond angle between beads *i*, *j* and *k*, and $\kappa$ the bending rigidity of the chain.

We parametrize the simulations to match the experimental polymers. Both donor and acceptor moieties in our chains have a dimension of approximately 1 nm, we thus set the bead size *σ* = 1 nm. The persistence length *l*_*p*_, of our donor chains, copolymers of dioctyl fluorene and either benzothiadiazole (*P*1) or phenyl (*P*2), is unknown. However, these data are known for pure homopolymer poly(dioctyl fluorene), measured to be *l*_*p*_ ~ 7 nm^73^, which is stiff due to the hairy-rod architecture, while alternating copolymers of equimolar mixtures of dioctyl fluorene and our acceptor DTBT, is estimated at *l*_*p*_ ~ 2 nm^74^. We estimate that for the non-stoichiometric copolymers we use here, the effective persistence length will be approximately 4 nm. Within our simulation model, it is known that *l*_*p*_/*σ* ≈ *κ*/*k*_*B*_*T*, we thus choose *κ*/*k*_*B*_*T* = 4. A chain of given degree of polymerisation was initialized and equilibrated extensively, after which 1000 statistically-independent snapshots of chain conformations were collected.

To go from chain conformations, sampled via Brownian Dynamics simulations, to the intrachain energy transfer efficiency, we use a recently developed coarse-grained theoretical approach^17^, which was shown to predict Förster resonant energy transfer (FRET) in polyfluorenes with good accuracy. We note that also excitonic processes, that occur along the semiconducting backbone, occur. In these simulations, for the sake of simplicity, we chose to focus solely on energy transfer through the dieelectric medium; previous work from our group has shown that this is a reasonable approximation^17^.

Since the semiconducting chain is composed of many donors and several acceptors, the total energy transfer efficiency can be written as:

7 $$E=\frac{1}{{n}_{D}}\,\sum _{j}^{nD}\,(\frac{{\sum }_{i}^{{n}_{A}}\,{(\frac{{r}_{F}}{{r}_{ij}})}^{6}}{1+{\sum }_{i}^{{n}_{A}}\,{(\frac{{r}_{F}}{{r}_{ij}})}^{6}})$$

in which *n*_*D*_ and *n*_*A*_ are the number of donor and acceptor beads in the chain respectively, with *N* = *n*_*D*_ + *n*_*A*_, *r*_*ij*_ the distance between donor *i* and acceptor *j*, and *r*_*F*_ the Förster radius, defining the distance where *E* = 0.5 for a single donor-acceptor pair. For a derivation of this result we refer to previous work^17^.

For each chain length, 1000 independent chain configurations were obtained using the BD simulations. To mimic the experimental scenario of random incorporation of the monomers from the reaction mixture, we randomly assign beads a donor or acceptor identity, drawing from a binomial distribution to ensure that over the ensemble of chains, the ratio of donor to acceptor beads is identical to the experiments. For each chain conformation, we generate 10 random donor-acceptor configurations, and compute *E* using Eq. 7. The calculated ensemble-averaged transfer efficiency is the mean over all 10 000 conformations generated as such for each chain length.

## Supplementary information

Supplementary information
