# Supplementary material for: Chain length-dependent luminescence in acceptor-doped conjugated polymers
Source: Sci Rep. 2019 Aug 2;9:11217. doi: 10.1038/s41598-019-47537-2 (PMC6677785; doi:10.1038/s41598-019-47537-2)
Supplement: Supplementary file 1 — Supplementary information [file 41598_2019_47537_MOESM1_ESM.pdf]

Chain length-dependent luminescence in acceptor-doped conjugated polymers

Pieter van der Scheer, Ties van de Laar, and Joris Sprakel*

Physical Chemistry and Soft Matter, Wageningen University and Research, Stippeneng 4, 6708WE, Wageningen, the Netherlands

E-mail: joris.sprakel@wur.nl

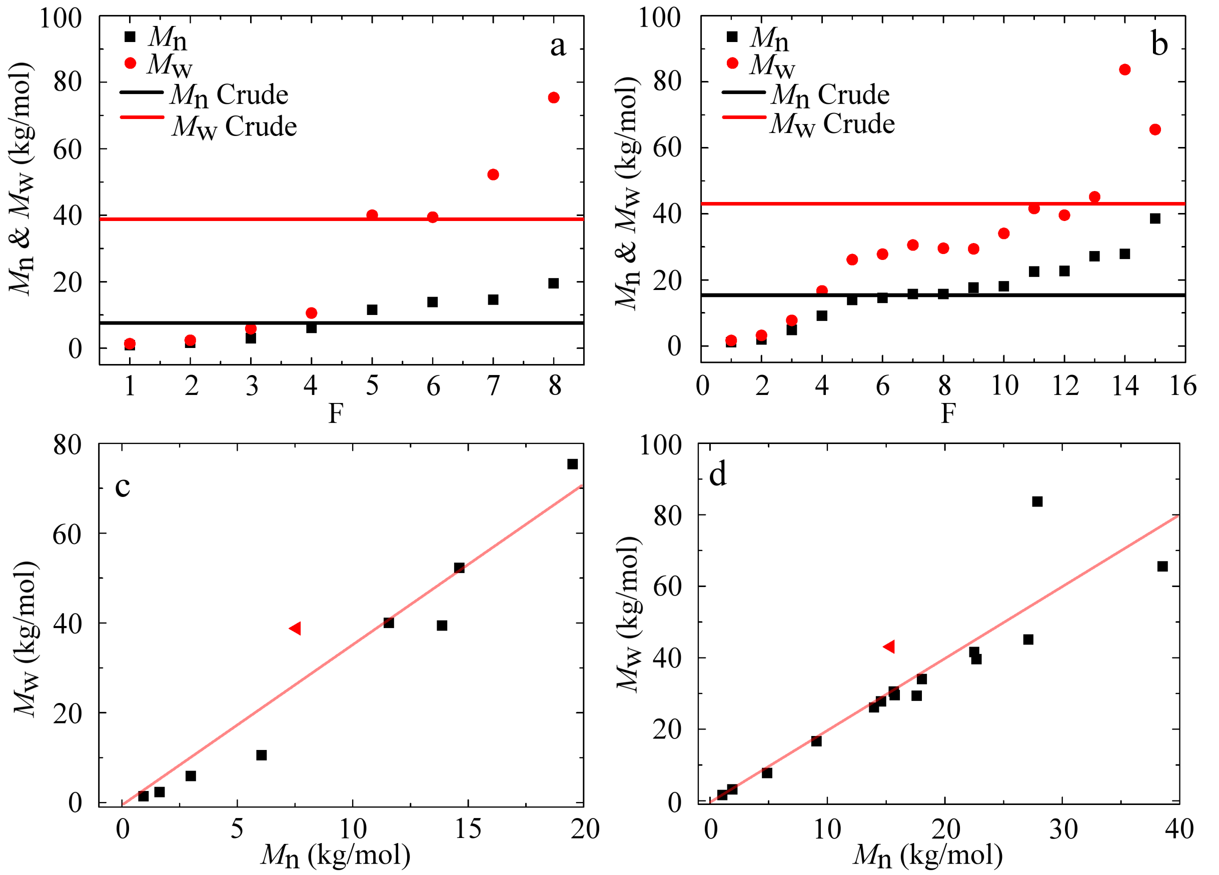

Figure 1: a-b) Molecular weights of $P1$ (a) and $P2$ (b) as a function of fraction number (see SI for details); average molecular weight of the crude products is indicated as solid lines. c-d) M_w as a function of M_n for the crude (red triangles) and different sub-fractions (black squares) for $P1$ (c) and $P2$ (d). The slope of the linear fit (solid red line) indicates the average polydispersity index.

Fractionation

Method

All crude polymer batches are purified from monomers, reaction by-products and residual catalyst, by Soxhlet extraction against methanol and acetone. This clean but polydisperse crude product is subsequently fractionated using solvent-gradient Soxhlet fractionation. Product is extracted in several steps, from low to high molecular weight fractions, based on chain-length dependent changes in solubility, which are particularly pronounced for the aromatic semiconducting polymers under study here. To do so, we gradually increase

the solvent quality in each subsequent extraction cycle. For each fraction, we isolate the fraction by precipitation in methanol and drying under vacuo. For the initial fractions, we use a combination of hexanes and THF, which have comparable boiling points and vapor pressures, but vastly different solvent qualities. Their respective boiling points are 68°C for hexane and 66°C for THF with vapor pressures of 17.60kPa for Hexanes and 17.59kPa for THF. For high molecular weight fractions, we use mixtures of THF and chloroform, ending with pure chloroform. Details on solvent mixtures and sequence are provided in the SI. For each new solvent, the product is extracted in the Soxhlet apparatus for at least 24h for the shorter fractions and 48h for the high molecular weight fractions. For two fractions of $P2$, we iterate the fractionation a second time, by exposing an initial medium M_w fraction, to a second round of Soxhlet extraction with smaller steps of the THF-hexane mixtures.

Data

By mixing solvents of similar boiling point and vapor pressure, but with vastly different solvent qualities for the conjugated polymers, we are able to fine tune the number of fractions into which the crude product is divided. For $P2$, we initially observed a diminished separating effect for the higher molecular weight fractions. This was most likely caused by shorter chains that were physically trapped within the powder in the Soxhlet thimble, being released together with longer chains as the solvency was increased. We improved upon this, by a secondary fractionation of the initial fractions (Table 1 and 2 SI) using a similar approach. This illustrates that the Soxhlet fractionation approach, while more feasible experimentally as compared to preparative GPC approaches to obtain sufficient material, is sensitive to the physical properties of the crude powders. Fine powders, loosely packed into the thimble, ensure maximum transport of species during the extraction.

This approach gives us access to a set of fractions of increasing average molecular weight (figure 3a-b), all derived from the same synthesis reaction, and thus featuring identical average chemical composition. When plotting M_w against M_n (SI figure3c-d) for both polymers

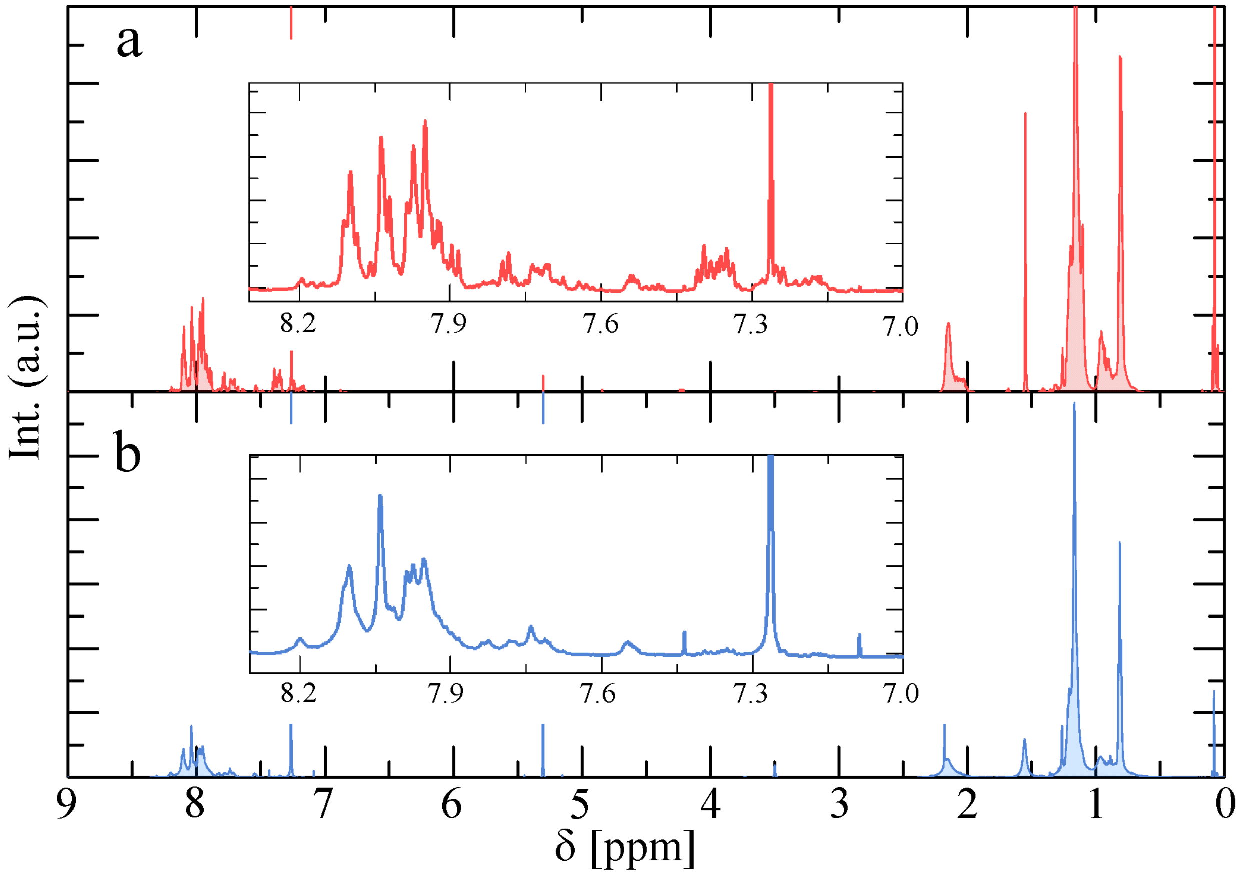

Figure 2: a-b)^{1}HNMR spectrum of P1-F2 & P1-F8 in $CDCl_3$

we can see the polydispersity, given as M_w/M_n, thus the slope of the curve in figure 3c-d, is improved for all but one fraction with respect to the crude product.

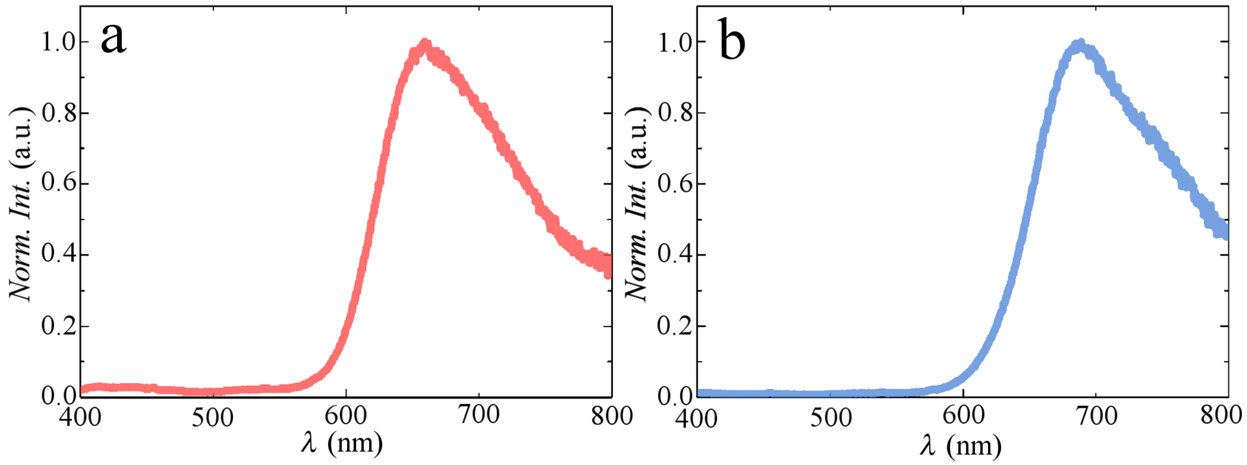

Figure 3: a-b)Solid state fluorescence emission spectra of P1-F1 & P1-F8. Drop-casted on quartz and excited at 370nm

P1				
Solvent	Mn (kg/mol)	Mw (kg/mol)	PDI	Fraction number
Crude	7.57	38.78	5.13	na
Acetone	0.93	1.36	1.46	1
Hexanes	1.63	2.36	1.44	2
Hexanes:THF (80:20)	2.99	5.90	1.97	3
Hexanes:THF (50:50)	6.05	10.56	1.74	4
THF	11.56	40.04	3.46	5
THF:$CHCl_3$ (90:10)	14.61	52.24	3.56	7
THF:$CHCl_3$ (70:30)	13.87	39.41	2.83	6
$CHCl_3$	19.52	75.38	3.86	8

Table 1: Soxhlet extraction solvents and GPC data for *P*1

P2				
Solvent	Mn (kg/mol)	Mw (kg/mol)	PDI	Fraction number
Crude	15.37	43.06	2.80	na
Acetone:Hexanes (50:50)	1.06	1.64	1.54	1
Hexanes:THF(80:20)	1.90	3.19	1.68	2
Hexanes:THF(60:40)	4.87	7.75	1.59	3
Hexanes:THF(40:60)	18.05	34.07	1.89	10
Hexanes:THF(55:45)	9.08	16.64	1.83	4
Hexanes:THF(50:50)	17.61	29.40	1.67	9
Hexanes:THF(45:55)	27.12	45.13	1.66	13
Hexanes:THF(40:60)	38.55	65.53	1.7	15
$CHCl_3$	14.57	27.76	1.90	6
Hexanes:THF(20:80)	15.72	29.56	1.88	8
Hexanes:THF(10:90)	15.64	30.53	1.95	7
THF	22.51	41.60	1.85	11
Hexanes:THF(15:85)	22.70	39.59	1.74	12
Hexanes:THF(5:95)	27.88	83.67	3.00	14
THF:$CHCl_3$(1:1)	13.97	26.10	1.87	5

Table 2: Soxhlet extraction solvents and GPC data for $P2$
